# Supplementary material for: Content-rich biological network constructed by mining PubMed abstracts
Source: BMC Bioinformatics. 2004 Oct 8;5:147. doi: 10.1186/1471-2105-5-147 (PMC528731; doi:10.1186/1471-2105-5-147)
Supplement: Additional File 2 — The original results of the above study (non-essential files are deleted to keep the file size under the limit set by BMC bioinformatics). [file 1471-2105-5-147-S2.bz2 › chilibotAdditionalFile2/dip40/373ID7680645E8296/html/FGF1_FGFR4.html]

 


 **FGF1** and **FGFR4** 
  
Found 127 abstracts in PubMed, retrieved 40.  
 

 What does Google say? 
 PDF only 
| .edu only 

---

**Interactive relationship** (e.g. stimulation, inhibition, etc)

**Neutral relationship**- We show here that oligosaccharides shorter than 8 sugar units support activation of FGFR2 IIIb by FGF 1  [ **FGF1** ]  and interaction of  **FGFR4**  with FGF 1  [ **FGF1** ] .  Ref: 11714710 J Biol Chem, 2002
- We conclude that  **FGFR4**  mediates FGF  [ **FGF1** ]  19 signaling to the PRL promoter.  Ref: 12169442 Am J Physiol Endocrinol Metab, 2002
- **FGFR4**  transduction cascades were differently activated by  **FGF1**  and FGF2.  Ref: 11336793 Biochim Biophys Acta, 2001
- RT PCR showed that FGFR1IIIc, FGFR2IIIc and  **FGFR4**  were the FGF  [ **FGF1** ]  8 receptors expressed in normal ovaries and in ovarian tumors.  Ref: 11072239 Int J Cancer, 2000
- We show that FGF  [ **FGF1** ]  19, which selectively binds  **FGFR4** , can induce prolactin PRL but not growth hormone expression.  Ref: 12169442 Am J Physiol Endocrinol Metab, 2002
- CONCLUSIONS These results suggest a paracrine autocrine modulation of epithelial and stromal cells of fibroadenomas through an aFGF  [ **FGF1** ]   **FGFR4**  interaction.  Ref: 11271786 J Clin Pathol, 2001
- The unique localization patterns of FGF6 and  **FGFR4**  may reflect specific roles for these members of the FGF  [ **FGF1** ]  signaling complex during myogenesis in adult skeletal muscle.  Ref: 10898801 J Histochem Cytochem, 2000
- ... FGF  [ **FGF1** ]  2 binds well to FGFR1, FGFR2, and  **FGFR4**  but interacts poorly with KGFR.  Ref: 10950949 J Biol Chem, 2000
- The FGF  [ **FGF1** ]  8 isoforms preferentially activate the receptors FGFR1IIIc, FGFR2IIIc, FGFR3IIIc and  **FGFR4** .  Ref: 11072239 Int J Cancer, 2000
- Strong  **FGFR4**  immunoreactivity was found in stromal fibroblasts, which were also weakly positive for aFGF  [ **FGF1** ] . aFGF  [ **FGF1** ]  mRNA was detected in epithelial cells and in some stromal fibroblasts.  Ref: 11271786 J Clin Pathol, 2001
- **FGF1** , FGF7, FGF9, FGF10, FGF11, and FGF18 were also expressed in the pancreas at varying time points and levels, as well as FGF  [ **FGF1** ]  receptor forms FGFR1b, FGFR1c, FGFR2b, FGFR2c, FGFR3b, and  **FGFR4** .  Ref: 12666204 Dev Dyn, 2003
- FGF  [ **FGF1** ]  19 also stimulated MAPK activation, an effect that was abrogated by a soluble dominant negative dn form of  **FGFR4** .  Ref: 12169442 Am J Physiol Endocrinol Metab, 2002
- We show that inhibition of  **FGFR4**  signal results in an arrest of muscle progenitor differentiation, which can be rapidly reverted by the addition of exogenous FGF  [ **FGF1** ] , rather than a modification in their proliferative capacities.  Ref: 12223412 Development, 2002

**Non-interactive relationship** (e.g. studied together, co-existance, homology, etc.)

- Expression of acidic fibroblast growth factor  [ **FGF1** ]  aFGF  [ **FGF1** ]  and fibroblast growth factor receptor 4  [ **FGFR4** ]   **FGFR4**  in breast fibroadenomas.  Ref: 11271786 J Clin Pathol, 2001
- The phosphatidylinositol 3 kinase inhibitors wortmannin and LY294002 only prevented the early ERK2 phosphorylation triggered by FGF2  **FGFR4**  but not by  **FGF1**   **FGFR4** .  Ref: 11336793 Biochim Biophys Acta, 2001
- Requirement for C terminal end of fibroblast growth factor receptor 4  [ **FGFR4** ]  in translocation of acidic fibroblast growth factor  [ **FGF1** ]  to cytosol and nucleus.  Ref: 10769213 J Cell Sci, 2000
- Formalin fixed, paraffin embedded normal tissues and 60 well characterized GEP endocrine tumors were immunostained using specific antibodies directed against various GEP hormones, aFGF  [ **FGF1** ] , FGFR1, FGFR2, FGFR3, and  **FGFR4** .  Ref: 11759058 Appl Immunohistochem Mol Morphol, 2001
- The aim of this study was to evaluate the distribution of aFGF  [ **FGF1** ]  and  **FGFR4**  in specific cell types of fibroadenomas to understand their possible role in the growth of these breast lesions.  Ref: 11271786 J Clin Pathol, 2001
- This provides an initial description of the expression in the developing skull of the genes encoding the secreted molecules BMP 2, BMP 4, BMP 7, the transmembrane FGF  [ **FGF1** ]  receptors FGFR 1, FGFR 2, FGFR 4  [ **FGFR4** ] , the transcription factors Msx1, Msx2, and Twist, as well as Goosecoid and the early membranous bone differentiation marker osteopontin.  Ref: 12710966 Dev Biol, 2003
- The ability of COS cells to bind and internalise acidic fibroblast growth factor  [ **FGF1** ]  aFGF  [ **FGF1** ]  was studied after transient transfection of the cells with wild type and mutated fibroblast growth factor receptor 4  [ **FGFR4** ] .  Ref: 10769213 J Cell Sci, 2000
- We also used RT PCR to analyze the expression patterns of the four tyrosine kinase FGF  [ **FGF1** ]  receptors FGFR1  **FGFR4**  and of the HGF receptor c met in the myofiber cultures.  Ref: 10898801 J Histochem Cytochem, 2000
- Other specific PTKs are also frequently overexpressed, including KDR and fibroblast growth factor receptor 4  [ **FGFR4** ]  FGF  [ **FGF1** ]  R4, while, interestingly, yet others, such as KIT and FES, are consistently downregulated in melanoma cell lines.  Ref: 11095400 Melanoma Res, 2000
- This soluble  **FGFR4**  isoform sFGFR4 abrogates the effect of FGF 1  [ **FGF1** ]  induced MAPK phosphorylation and PRL gene activation.  Ref: 11549253 Biochem Biophys Res Commun, 2001
- We show that the PAC1 sublines that undergo a more robust transition into the skeletal muscle phenotype also express significantly higher levels of the insulin like growth factor IGF1 and IGF2 genes and of FGF  [ **FGF1** ]  receptor 4  **FGFR4**  gene.  Ref: 10950875 J Histochem Cytochem, 2000
- Constitutive overexpression of FGF  [ **FGF1** ]  8b in MCF 7 cells down regulated FGF  [ **FGF1** ]  8b binding receptors FGF  [ **FGF1** ]  receptor FGFR 1IIIc, FGFR2IIIc, and  **FGFR4**  found to be expressed in these cells.  Ref: 11358849 Cancer Res, 2001
- In order to investigate further the role of FGF  [ **FGF1** ]  signalling during mouse calvarial bone and suture development, we have performed detailed expression analysis of the splicing variants of Fgfr1 Fgfr3 and  **Fgfr4** , as well as their potential ligand Fgf2.  Ref: 10751173 Development, 2000
- Thus, we examined whether FGF  [ **FGF1** ]  19 might regulate its own receptor through the Ets Ik element in the  **FGFR4**  promoter.  Ref: 12169442 Am J Physiol Endocrinol Metab, 2002
- Acidic fibroblast growth factor  [ **FGF1** ]  aFGF  [ **FGF1** ]  is a well known fibroblast activator, which acts through four specific cell surface receptors, among which, fibroblast growth factor receptor 4  [ **FGFR4** ]   **FGFR4**  is highly specific.  Ref: 11271786 J Clin Pathol, 2001
- In contrast, Ets enhanced FGF  [ **FGF1** ]  19 induced PRL activation but failed to confer an effect for FGF  [ **FGF1** ]  19 on the  **FGFR4**  promoter.  Ref: 12169442 Am J Physiol Endocrinol Metab, 2002
- These findings represent the first description of an endogenous soluble C terminally truncated  **FGFR4**  isoform with FGF  [ **FGF1** ]  modulatory properties.  Ref: 11549253 Biochem Biophys Res Commun, 2001
- RESULTS Immunoreactivity for aFGF  [ **FGF1** ]  and  **FGFR4**  was seen in epithelial cells, but it was lacking in myoepithelial cells of both normal tissues and fibroadenomas.  Ref: 11271786 J Clin Pathol, 2001
- The authors focused on FGFR 4  [ **FGFR4** ]  expression in astrocytoma because the FGF  [ **FGF1** ]  expression increases as the tumor malignancy progresses.  Ref: 11958417 Neurol Res, 2002
- FGF  [ **FGF1** ]  receptor FGFR 1 and FGFR 4  [ **FGFR4** ]  were detectable in the mouse submandibular glands and carcinomas.  Ref: 11271631 J Oral Pathol Med, 2001
- METHODS Formalin fixed and paraffin wax embedded tissues from 15 fibroadenomas and peritumoral normal breasts were investigated for the expression of aFGF  [ **FGF1** ]  and  **FGFR4**  using immunohistochemistry.  Ref: 11271786 J Clin Pathol, 2001
- We demonstrate that FXR directly regulates expression of fibroblast growth factor 19 FGF  [ **FGF1** ]  19, a secreted growth factor that signals through the  **FGFR4**  cell surface receptor tyrosine kinase.  Ref: 12815072 Genes Dev< MedlineTA>Genes Dev, 2001
- Early phosphorylation of ERK2 was blocked by the dominant negative form of growth factor bound protein 2 and Ras, for  **FGF1**   **FGFR4**  and FGF2  **FGFR4** .  Ref: 11336793 Biochim Biophys Acta, 2001
- In chick embryos, most if not all, replicating myoblasts present within the skeletal muscle masses express high levels of the FGF  [ **FGF1** ]  receptor FREK  **FGFR4** , suggesting an important role for this molecule during myogenesis.  Ref: 12223412 Development, 2002
- Stimulation of FGFR1 by  **FGF1**  or FGF2 and  **FGFR4**  by  **FGF1**  induced a sustained phosphorylation of extracellular signal regulated protein kinase 2 and meiosis reinitiation.  Ref: 11336793 Biochim Biophys Acta, 2001
- Of the four fibroblast growth factor FGF  [ **FGF1** ]  receptor genes expressed in adult liver,  **FGFR4**  is expressed specifically in mature hepatocytes.  Ref: 12466116 Am J Pathol, 2002
- We report the identification of a novel pituitary tumor derived, N terminally truncated isoform of FGF  [ **FGF1** ]  receptor 4 ptd  **FGFR4** .  Ref: 11781352 J Clin Invest, 2002
